# L'ostéotomie fémorale de varisation par ouverture externe pour les gonarthroses fémoro-tibiales latérales sur genuvalgum idiopathique: étude rétrospective de 10 cas

**DOI:** 10.11604/pamj.2017.28.22.12014

**Published:** 2017-09-13

**Authors:** Asma Ben Cheikh, Mahmoud Ben Maitigue, Karim Masmoudi, Thabet Mouelhi, Nader Naouar, Yamen Grissa, Karim Bouattour, Walid Osman, Mohamed Laziz Ben Ayeche

**Affiliations:** 1 Service d'Hygiène Hospitalière, CHU Farhat Hached de Sousse, Faculté de Médecine de Sousse, CP 4000, Sousse, Tunisie; 2 Service de Chirurgie Orthopédique et Traumatologique, Faculté de Médecine de Sousse, CHU Sahloul, Hammam Sousse CP 4011, Sousse, Tunisie; 3 Faculté de médecine de Sousse, Avenue Mohamed Karoui, CP 4000, Sousse, Tunisie

**Keywords:** Genu valgum, ostéotomie, fémur, gonarthrose, Genu valgum, osteotomy, femur, gonarthrosis

## Abstract

L'ostéotomie fémorale de varisation est un traitement conservateur des gonarthroses uni-compartimentales externes qui reste de pratique peu courante et dont les résultats sont peu étudiés. L'objectif de ce travail était d'évaluer les résultats cliniques et radiologiques de l'ostéotomie fémorale de varisation chez les sujets présentant une gonarthrose fémoro-tibiale externe sur genou valgum idiopathique répertoriés sur une période de 21 ans(de 1992 à 2013) au service d'Orthopédie du CHU Sahloul à Sousse. L'évaluation clinique des patients a été faite à partir du score IKS (International Knee Society). L'évaluation radiologique a été basée sur un bilan pré opératoire et un bilan au dernier recul. L'étude portait sur une série de 9 patients (et 10 genoux) dont l'âge moyen était de 45,2 ans avec un sex ratio de 0,5. Le recul moyen était de 99 mois. Le score genou moyen est passé de 48,4 points en préopératoire à 73,5 points au dernier recul avec une amélioration statistiquement significative (p<10-3). Le score fonctionnel moyen s'est amélioré d'une façon significative avec une valeur préopératoire de 49,5 points et une valeur 72 pointsau dernier recul. La correction finale a permis de réduire le valgus à une moyenne de 3,7° pour une valeur pré-opératoire de 14°. Cette étude, ainsi que l'analyse de la littérature, nous ont permis de déduire que l'ostéotomie fémorale de varisation est l'indication de choix dans les genuvalgum invalidant d'origine fémorale, en l'absence d'arthrite rhumatismale, de surcharge pondérale, d'arthrose fémoro-tibiale interne ou fémoro-patellaire sévère.

## Abstract (en)

Femoral varus osteotomy is a conservative treatment for external single-compartment gonarthrosis. This surgical procedure is little used and outcomes are little studied.This study aimed to assess the clinical and radiological results of femoral varising osteotomy in subjects with external femorotibial gonarthrosis associated with idiopathic genu valgum whose data were recorded over a period of 21 years (1992- 2013) in the Department of Orthopedics at Sahloul University Hospital, Sousse. The clinical evaluation of patients was performed using the IKS score (International Knee Society). Radiological assessment was based on pre-operative work-up and final follow-up assessment. We here report a case series of 9 patients (and 10 knees) whose average age was 45.2 years, with a sex ratio of 0.5. Mean follow-up was 99-months. Average knee score ranged from 48.4 points preoperatively to 73.5 points at the final follow-up assessment, with a statistically significant improvement (p<10-3). Medium functional score significantly improved, with a preoperative value of 49.5 points and a value of 72 points at the final follow-up assessment. Final correction helped to reduce valgus condition with an average of 3.7° and a preoperative value of 14°. This study, as well as the analysis of literature, indicates that femoral varising osteotomy is the treatment of choice for invalidating genu valgum of femoral origin, without any rheumatoid arthritis, overweight, internal femorotibial nor severe femoropatellar arthritis.

## Introduction

La gonarthrose constitue une des localisations préférentielles de la maladie dégénérative du cartilage [1]. Il s'agit d'une maladie évolutive. Non traitée, le pronostic fonctionnel est sombre à cause de la destruction articulaire. Par conséquent, elle est génératrice d'incapacité voire d'un handicap chez les personnes dès l'âge de 45 ans [1]. Plusieurs études ont montré que la désaxation du genou au plan frontal est un facteur important dans la genèse et l'évolution de la gonarthrose fémoro-tibiale uni-compartimentale [2]. Actuellement, le traitement médical de la gonarthrose n'est que symptomatique et nous sommes souvent amenés à proposer un traitement chirurgical à nos patients après épuisement du traitement médical et physique.

Malgré les progrès réalisés, le traitement prothétique total ou partiel du genou reste toujours insuffisant pour supporter durablement une activité importante chez des patients jeunes et actifs. Sur des bases biomécaniques, les ostéotomies étaient proposées pour une population jeune, valide et active [3]. Elles s'inscrivent dans le cadre des traitements conservateurs qui visent à ralentir l'évolution de la gonarthrose. Les ostéotomies pour déformation en valgus sont beaucoup moins fréquentes que celles pour déformation en varus comme en témoignent les séries de la littérature qui sont moins nombreuses [4, 5].

Bouillet and Van Gayer, en 1961, Coventry en 1973 étaient parmi les premiers auteurs qui avaient proposé une ostéotomie fémorale de varisation (OFV) [6, 7]. Langlais avait proposé la technique de l'ostéotomie fémorale distale d'ouverture externe jugée plus simple que les autres techniques de fermeture interne [6, 8]. Les résultats au moyen et au long cours de ces ostéotomies ont été décrits par plusieurs auteurs [4, 6]. Cette technique était adoptée au service d'Orthopédie du CHU Sahloul à Sousse. Elle reste toujours peu étudiée et de pratique peu fréquente. C'est dans ce cadre que nous avons mené cette étude rétrospective dont l'objectif était d'évaluer les résultats cliniques et radiologiques de l'ostéotomie fémorale de varisation chez les sujets présentant une gonarthrose sur genu valgum d'origine idiopathique.

## Méthodes

### Lieu d'étude

Cette étude a été menée au C.H.U. Sahloul de Sousse lequel est composé de 15 services médicaux, 10 services chirurgicaux, 8 blocs opératoires, 3 laboratoires et doté d'une capacité hospitalière de 583 lits en 2014.

### Population et type d'étude

Il s'agissait d'une étude rétrospective descriptive portant sur douze patients (soit 14 genoux) ayant une gonarthrose fémoro-tibiale externe sur genu valgum idiopathique et opérés par OFV, colligés sur une période de 21 ans (de 1992 à 2013) dans le service d'Orthopédie du CHU Sahloul à Sousse. Ont été inclus dans ce travail seulement les patients ayant un recul minimum d'un an avec des renseignements cliniques et radiologiques suffisants et avec une uniformité de la technique chirurgicale. Les patients qui ont un genu valgum non idiopathique n'ont pas été inclus dans cette étude.

### La collecte des données

Au début, nous avons identifié tous les cas ayant une gonarthroses fémoro-tibiale externe sur genu valgum d'origine idiopathique et opérés par OFV durant une période de 21 ans (de 1992 à 2013). Dans un deuxième temps, nous avons essayé de convoquer tous ces patients. Ceux qui ont répondu à notre convocation ont été examinés et ont eu un bilan radiologique complet de contrôle qui a servi à l'évaluation des résultats au dernier recul. Ceux qui n'ont pas répondu à notre convocation ont été évalués sur les données de leurs dossiers au dernier recul. Le recueil des données a été fait sur une grille préétablie comportant les données anamnestiques, cliniques et radiologiques pré et postopératoires ainsi que la technique chirurgicale pratiquée pour ces patients: 1) **Les données épidémiologiques** suivantes ont été recueillies du dossier médical: l'âge, le genre, le côté atteint. 2) **Les données cliniques pré et postopératoires** ont été évalué par les scores genou et fonctionpré opératoire et au dernier recul, adoptés par la société internationale de chirurgie du genou («International Knee Society» ou «IKS»). Ce score a été décrit par Insall [9]. Il a été utilisé afin d'évaluer l'état clinique pré et post opératoire des patients. Ce score est divisé en deux parties: 1) **Le score genou:** sur 100 points (permet d'évaluer le genou concerné). 2) **Le score fonction:** sur 100 points (permet d'évaluer l'état clinique et fonctionnel du patient).

Ainsi, nos patients ont été classés en quatre groupes selon les valeurs du score IKS (Tableau 1) [9]. Mis à part le score «IKS», nous avons recherché un syndrome fémoro-patellaire lors de l'examen du genou. Le bilan radiologique comportait des radiographies des deux genoux de face et de profil, une incidence fémoro-patellaire et une radiographie télémétrique des deux membres inférieurs préopératoire et au recul.

**Tableau 1:** La classification des patients selon le score genou et fonction de l’«IKS»

Groupes	Score genou «IKS»	Score fonction «IKS»
(A)Excellent	85-100	85-100
(B)Bon	70-84	70-84
(C) Moyen	60-69	60-69
(D) Mauvais	0-59	0-59
(E)Echec	Remplacement prothétique	Remplacement Prothétique

**Figure 1:**
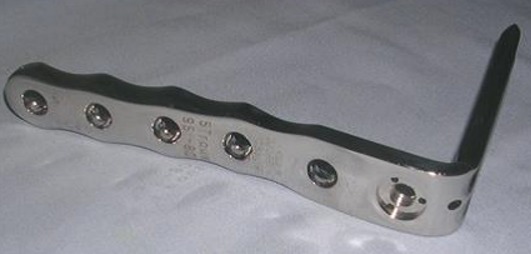
La lame-plaque Strélizia 95

**Figure 2:**
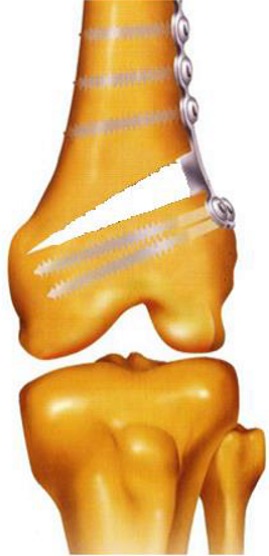
La technique de l'ostéotomie fémorale de varisation par ouverture externe sans interposition de griffon

**La technique chirurgicale:** Consiste en une ostéotomie d'ouverture externe du fémur, bâillante, sans greffe osseuse, utilisant une lame plaque de type Strélizia 95°(Figure 1 et Figure 2) [10]. L'ostéotomie fémorale d'ouverture externe est bien codifiée et répond dans notre service au même schéma: une bonne position de la lame (position épiphysaire à 25 mm de l'interligne) et une ostéotomie à 50 mm de l'interligne. La lame plaque permet une correction automatique avec impaction interne.

**Analyse statistique des données:** La saisie et l'analyse des données ont été effectuées sur le logiciel SPSS 20. Les variables quantitatives ont été résumées sous forme de moyennes plus ou moins leurs écart-types. Les variables qualitatives ont été résumées par des fréquences absolues et relatives. Le test de Chi2 (χ^2^) ou le test exact de Fisher étaient utilisés pour les variables qualitatives (si les conditions d'applications le permettaient). Pour les variables quantitatives, la comparaison entre les différentes variables a été faite par le test t de Student sur séries appariées. Des valeurs de p inférieures à 0,05 ont été considérées comme statistiquement significatives.

**Considérations éthiques:** Outre l'information et le consentement oral des participants, cette étude n'a pas fait l'objet de considérations éthiques particulières.

## Résultats

Nous avons retenu 9 dossiers répondant aux critères d'inclusion (soit 10 genoux opérés). Le recul moyen était de 99 mois avec des extrêmes allant de 12 mois à 276 mois. Il s'agissait de 6 femmes et 3 hommes dont l'âge moyen à l'intervention était de 45, 2 ± 10,5 ans (entre 20 ans et 57 ans). Le sexe ratio était de 0,5. Un seul patient a été opéré des deux côtés. Le côté droit était concerné dans 8 cas et le côté gauche dans 2 cas.

Le délai entre le début de la symptomatologie et l'intervention variait de 1 à 15 ans. Le score genou moyen selon l' «IKS» est passé de 48,4 points en préopératoire à 73,5 points au dernier recul avec des extrêmes allant de 42 à 94 points (p<10^-3^). De même, le score fonction moyen est passé de 49,5 points en préopératoire à 72 points au dernier recul avec des extrêmes allant de 0 à 100 points (p=0,04). En ce qui concerne les scores élémentaires selon l'IKS: nous avons constaté une amélioration significative de la douleur, avec un score élémentaire moyen qui est passé de 19 points en pré-opératoire à 41 points au dernier recul (p<10^3^) (Tableau 2). L'amplitude articulaire a augmenté de manière non significative, avec un score mobilité moyen de 23,8 points au dernier recul, pour une valeur préopératoire de 23,6 points (p=0,7). Le périmètre de marche moyen s'est amélioré d'une façon significative en passant de 22 points en préopératoire à 36 points au dernier recul (p=0,04) (Tableau 2). Le score relatif à la pratique des escaliers est passé de 26,5 points à 36,5 points au dernier recul (p=0,08). L'évaluation objective des résultats est résumée dans le Tableau 3.

**Tableau 2:** Résultats cliniques: évolution du score douleur et du périmètre de marche après l’ostéotomie fémorale de varisation (N=10)

	Nombre de genoux	
	En Pré opératoire	En post opératoire
**Douleur**		
Aucune douleur	0	2
Douleur légère ou occasionnelle	0	6
Uniquement dans les escaliers	0	0
A la marche et dans les escaliers	5	2
Douleur modérée occasionnelle	0	0
Douleur modérée permanente	4	0
Douleur sévère	1	0
**Périmètre de marche**		
Périmètre illimité	0	4
Périmètre à 1000 mètres	1	2
Périmètre entre 500 et 1000 mètres	2	2
Périmètre de marche < 500 mètres	7	1
Périmètre de marche dans la maison	0	1
Marche impossible	0	0

**Tableau 3:** L’évaluation objective des résultats cliniques en postopératoire selon l’ «IKS» (Total=10)

Résultats	Nombre des patients	
	Score genou de l’IKS	Score fonction de l’IKS
Excellent	3	6
Bon	4	0
Moyen	1	2
Mauvais	2	2
Remplacement prothétique	0	0

**Figure 3:**
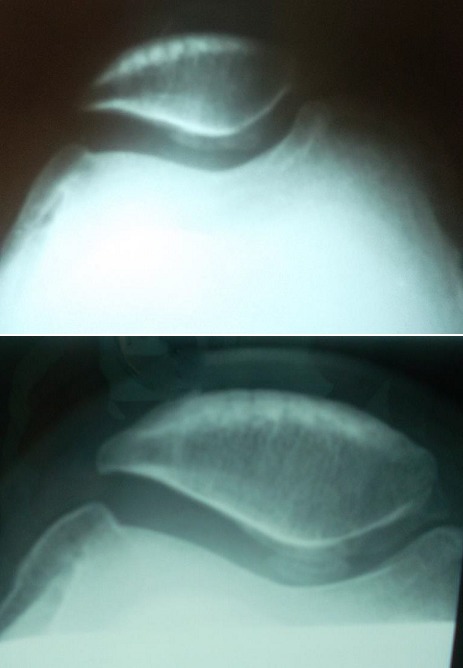
Radiographies fémoro-patellaires à 30° de flexion du genou droit, en pré-opératoire et au dernier recul chez un patient de notre série ayant une arthrose fémoro-patellaire associée

**Figure 4:**
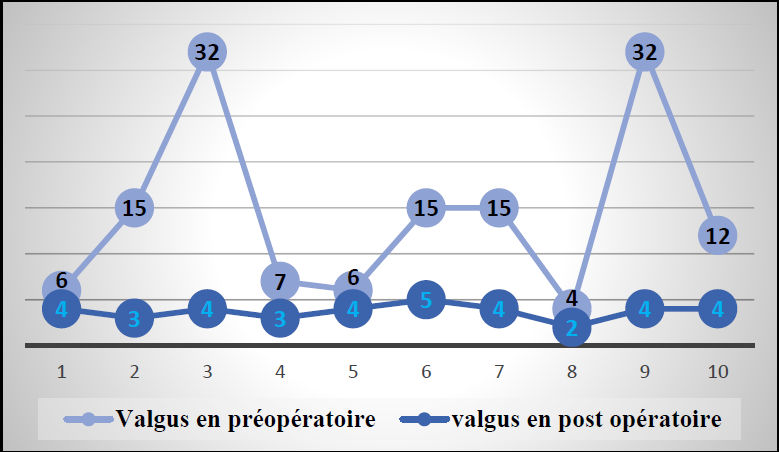
Résultats radiologiques: évolution du valgus en pré et en post opératoire (N=10, p=0,008)

**Figure 5:**
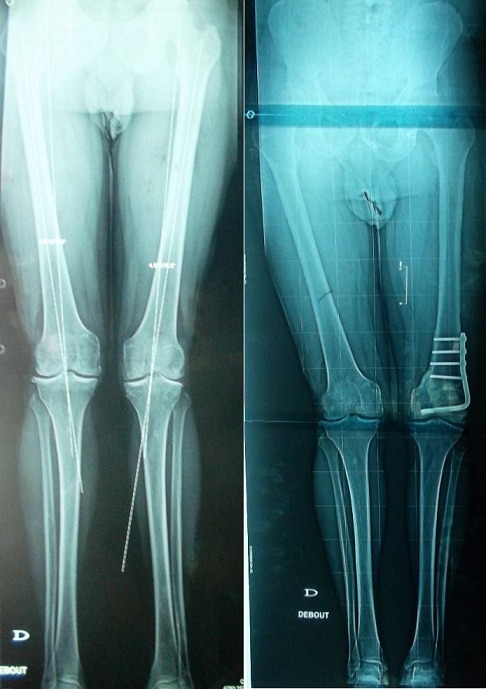
Ostéotomie fémorale de varisation du genou gauche, chez un patient âgé de 51 ans, ayant permis une réduction du valgus de 7° en préopératoire à 3° au dernier recul

Six patients avaient, avant l'intervention, une symptomatologie en rapport avec une atteinte de l'articulation fémoro-patellaire. Ils ont présenté une douleur élective à la palpation du bord externe de la rotule associée à un signe de Rabot et un signe de Zohlen positifs. Selon la classification d'Ahlback de l'arthrose fémoro-tibiale externe, 4 patients avaient un stade II, la moitié des patients avaient un stade III (cinq patients) et un seul patient avait un stade IV. Trois patients avaient une arthrose fémoro-patellaire externe (Figure 3). Les radiographies télémétriques des membres inférieurs nous ont permis de calculer les différents angles mécaniques du genou et de déterminer l'angle fémoro-tibial mécanique moyen de notre série qui était de 192,4° avec des extrêmes allant de 184° à 199°.La déviation angulaire était de 14,4° avec des extrêmes allant de 4° à 32°. L'évolution du valgus en pré et en post opératoire est représentée dans les Figure 4 et Figure 5.

La consolidation des ostéotomies a été obtenue chez tous les patients dans un délai moyen de 14 semaines avec des extrêmes allant de 12 à 20 semaines. Deux types de complications ont été notés: un retard de consolidation de l'ostéotomie chez une patiente (au 5ème mois post-opératoire), une saillie gênante interne de la lame nécessitant son ablation chez 3 patients. Au dernier recul, aucun patient n'avait nécessité le recours à un remplacement prothétique.

## Discussion

L'OFV d'ouverture externe est l'intervention chirurgicale effectuée pour le traitement de l'arthrose du compartiment fémoro-tibial latéral du genou, et pour la correction de la déformation fémorale en valgus. Cette procédure reste de pratique peu fréquente dont les résultats sont peu publiés vu la bonne tolérance de cette variété de gonarthrose [7, 11].

Ce travail constitue une des rares études de l'OFV sur la gonarthrose fémoro-tibiale latérale d'origine idiopathique. Dans une des séries de la littérature (Zarrouk 2010), 23 cas d'OFV sur genu valgum ont été répertoriés. Cependant, l'étiologie du genu valgum était diversifiée (cause traumatique, congénitale, séquelles de poliomyélite, dysplasie épiphysaire multiple) [11]. Et Par suite, notre étude est considérée comme la première enquête faite à Sousse et au CHU Sahloul. Au cours de notre étude, nous avons utilisé le score IKS qui représente un des scores les plus fiables et reproductibles dans l'évaluation de la fonction du genou. Il constitue également le score le plus utilisé dans les publications précédentes ce qui facilite l'étude comparative [12]. Le recul moyen de 99 mois, comparables aux études précédentes et l'unicité de la technique chirurgicale employée constituaient aussi des points forts de cette étude. Cependant, l'effectif restreint (9 patients et 10 genoux seulement) est considérée comme une limite de ce travail vu qu'il réduit les conclusions potentielles qui pourraient être tirées. Néanmoins, cet effectif réduit reste comparable aux séries de la littérature (Tableau 4) [4, 5, 7, 13–15]. Ilpeut être expliqué par la rareté de la déformation du genou en valgum, la bonne tolérance de l'arthrose fémoro-tibiale externe et le caractère rétrospectif de l'enquête.

**Tableau 4:** Les séries de la littérature sur l’ostéotomie fémorale de varisation

Auteurs	Age moyen (ans)	Effectif(patients/genoux)	SexeH/F	Recul (mois)	AFTmPré-op	AFTmPost-op	Type de matériel	Score fonction satisfaisant	Score genou satisfaisant
**Stahelin** (2000) **[15]**	57	19/21	10/9	60	192°	181,7°	Plaque AO	90%	-
**Marin Morales**(2000) **[4]**	55	17/19	5/12	78	196°	181°	Lame-plaque 95°	71%	94%
**Aglietti**(2000) **[5]**	50,5	14	4/10	108	197,5°	186°	-	77%	-
**Zilber**(2004) **[14]**	45	11	6/5	126	193°	182°	Lame-plaque	40%	90%
**Wang** (2006) **[13]**	53	30	2/28	95	198,2°	181,2°	Lame-Plaque 90°	83%	-
Backstein (2007) **[7]**	44,1	38/40	10/28	123	191,6°	181,2°	Lame-plaque 90°	60%	-
Zarrouk (2010) **[11]**	53	20/22	7/13	50	194,5°	181,5°	Lame-plaque 95°	80%	-
**Notre série**	**45,2**	**9/10**	**3/6**	**99**	**192,4°**	**184°**	**Lame-plaque 95°**	**60%**	**70%**

L'OFV fait partie intégrante du traitement conservateur du genuvalgum. Selon plusieurs études, ce type de traitement devrait être pratiqué à un âge inférieur à 65 ans [10, 13]. Marin Morales et Coll. mentionnaient que la tranche d'âge ayant les meilleurs résultats était comprise entre 50 et 70 ans [4]. Certains auteurs ont proposé un âge limite à 65 ans pour les OFV en insistant sur l'importance de l'âge physiologique et non chronologique [16]. Dans cette étude, l'âge moyen des patients au moment de l'intervention était de 45 ans.

Sans tenir compte du facteur âge et du facteur étiologie, nous avons comparé nos résultats cliniques avec ceux de la littérature: Marin Morales, Aglietti et Menchetti utilisaient les scores HSS (Hospital for specialsurgery) [17] ou IKS [9]. Ils ont trouvé des résultats bons et excellents dans 71 à 83% des cas [4, 5]. Dans notre série, parmi les 10 genoux opérés, 7 patients avaient un bon ou un excellent résultat selon la composante genou du score IKS (70%), et 6 patients avaient un bon ou un excellent résultat selon la composante fonctionnelle du score IKS (60%). A travers l'ensemble de ces constatations, nous avons pu déduire que les résultats de l'OFV étaient satisfaisants.

La déviation angulaire constituait un facteur pronostique très important. La majorité des auteurs s'accordent sur le fait qu'une déformation angulaire en valgus supérieure à 15° est considérée comme majeure [4, 6, 10, 18]. Au-delà de 20°, plusieurs facteurs vont influencer négativement le résultat au dernier recul du fait des difficultés opératoires de correction et de la tendance à la récidive de la déviation en raison de la laxité ligamentaire associée et du stade évolué de l'arthrose. Selon la littérature, les auteurs recommandaient l'ostéotomie pour les déviations angulaires supérieures à 12° [4, 6, 19].

Pour notre population, le valgus était en moyenne de 14,4° en préopératoire. La correction finale a permis de réduire, d'une manière significative le valgus à une moyenne de 3,7° avec des extrêmes allant de 2 à 5°. Selon l'étude menée par Zarrouk, le valgum moyen préopératoire était de 14,9° et l'OFV a permis de corriger le valgus d'une moyenne de 11,5° [11]. L'étude faite par Zilber a montré que le genu valgum moyen était de 12° en préopératoire. Le genu valgum au dernier recul était de 3° ce qui correspond à une correction moyenne de 10,5° [14].

L'angle de correction nécessaire pour soulager durablement une gonarthrose fémoro-tibiale externe reste controversé. Selon la littérature, cet angle variait entre 6° de varus et 10° de valgus [11, 18]. En effet, certains auteurs préconisent une hypo-correction et gardent un genu valgum de 2 à 4° [5, 6, 11, 18]. Au moment de la planification préopératoire, nous avons recommandé de garder un léger valgus résiduel de 2° à 3°, lequel a été recommandé auparavant par plusieurs auteurs [5, 6]. D'autres recommandaient une normo-correction ou une hyper correction du genu valgum [14]. Ceci a pour but de soulager le compartiment fémoro-tibial latéral et de prévenir la rechute de la déformation [16, 17].Cependant, selon certaines études précédentes, une correction excessive avec un genuvarum supérieur à 9° est génératrice de moins bons résultats [4]. La grande variabilité de l'angle de correction final du valgus dans la littérature montre que l'OFV est une procédure difficile à régler avec précision, et que l'angle de correction planifié en préopératoire n'était pas toujours atteint. Ceci pourrait s'expliquer par une perte de fixation ou par effondrement post opératoires des ostéotomies.

Les résultats des OFV dépendent du stade évolutif de l'arthrose. En effet, les résultats de cette procédure sont meilleures lorsque la gonarthrose est peu évoluée et uni-compartimentale. Les stades avancés (III, IV et V), peuvent retentir sur le résultat final des OFV. L'usure assez évoluée et la distension dans le compartiment ligamentaire constituent une source de défaut de correction et de récidive de la déformation [4, 11]. Dans notre série, 9 genoux sur 10 avaient des arthroses aux stades II et III. Sept parmi les neufs cas (77%) avaient un bon ou un excellent score genou de l'IKS. Concernant le score fonction, 8 cas parmi les neufs (88%) avaient un bon ou un excellent résultat. Selon l'étude faite par Antonescu, les résultats étaient meilleurs dans les gonarthroses peu évoluées uni-compartimentale stade I et II avec 85% de bons et de très bons résultats, contre 42% seulement pour les gonarthroses évoluées [2]. Ainsi, dans les gonarthroses évoluées, la limitation des mouvements articulaires et l'existence d'une laxité représentaient des facteurs de mauvais pronostic.

Le secteur de mobilité constitue un facteur d'indication chirurgicale et d'évaluation post-opératoire des résultats de l'OFV. Cette procédure peut engendrer une raideur articulaire surtout lorsqu'elle est associée à un geste articulaire (méniscectomie, libération de l'aileron rotulien…) ou à une immobilisation post-opératoire prolongée. Dans notre série, on a constaté une discrète amélioration du secteur de mobilité au dernier recul, qui était statistiquement non significative (p=0,7). D'après certains auteurs, l'OFV devrait être contre-indiquée en cas de flexion inférieure à 90° et de flessum supérieur à 15° [14, 18]. L'amélioration de la mobilité peut être mise sur le compte de la mobilisation précoce grâce à une ostéosynthèse solide et stable, ainsi que l'effet antalgique de l'ostéotomie [7, 18]. La laxité ligamentaire est un élément important à rechercher : selon certains auteurs, la présence d'une laxité frontale supérieure à 5 mm constituait une contre-indication à l'OFV du fait du risque de récidive de la déformation et de la persistance de la contrainte sur le compartiment fémoro-patellaire interne.

Parmi les autres facteurs pouvant influer sur le résultat final de l'OFV, est l'arthrose fémoro-patellaire. Le rôle de l'OFV pour le traitement de l'arthrose fémoro-patellaire externe reste controversé [11, 15]. Plusieurs auteurs se sont intéressés à l'étude de ce facteur. Pour Stahelin et Insall, la gonarthrose fémoro-patellaire avancée associée à une gonarthrosefémoro-tibiale externe sur genu valgum constituait une contre-indication à l'OFV [11, 15]. Sur le plan coronal, l'OFV altérait la biomécanique de l'articulation fémoro-patellaire en augmentant l'angle entre le quadriceps et le tendon rotulien ce qui permettrait de réduire la force qui traçait la rotule en latéral. Il préconisait d'associer avec l'OFV un avancement de la tubérosité tibiale antérieure [20]. Dermott a trouvé, après un suivi moyen de 99 mois, de bons résultats de l'OFV même en présence d'une arthrose fémoro-patellaire modérée [6]. L'étude faite par Ouadih a relevé une stabilisation de l'arthrose fémoro-patellaire dans 10 cas et une amélioration de l'interligne dans 6 cas [19]. Wang a montré une amélioration chez 7 parmi les 8 patients ayant une arthrose fémoro-patellaire sévère. Selon lui, une subluxation de la rotule et une arthrose fémoro-patellaire externe nécessiteraient une libération de l'aileron rotulien externe, tandis que la luxation habituelle de la rotule nécessiterait, en plus de la libération de l'aileron rotulien externe, un avancement et une transposition interne de la TTA [13]. Dans notre série, la libération de l'aileron rotulien externe a permis à trois patients de stabiliser l'arthrose fémoro-patellaire externe et d'améliorer leur symptomatologie.

## Conclusion

D'après l'analyse de cette série et de celles de la littérature, l'OFV est indiquée dans le genuvalgum invalidant d'origine fémorale chez des patients jeunes, actifs, sans surcharge pondérale importante, en l'absence d'arthrite rhumatismale du genou, de laxité frontale, d'arthrose fémoro-tibiale médiale et d'arthrose fémoro-patellaire sévère. La correction finale de la déformation ne devait pas dépasser 3° degrés de genu varum. La fixation de l'ostéotomie par une lame plaque sans greffe osseuse permet non seulement la rééducation immédiate mais aussi la consolidation du foyer de l'ostéotomie dans tous les cas. Dans ces conditions, l'OFV peut apporter des résultats satisfaisants à moyen et à long terme, surtout quand elle est pratiquée précocement au stade pré-arthrosique.

### Etat des connaissances actuelles sur le sujet

- L'OFV est le traitement de choix des gonarthroses uni-compartimentale fémoro-tibiale latérale;
- La gonarthrose fémoro-tibiale latérale sur genu valgum est une entité rare, souvent bien tolérée, qui nécessite rarement le recours à la chirurgie;
- Le résultat final de l'OFV dépend essentiellement du stade évolutif de l'arthrose et de l'importance de la déviation angulaire.

### Contribution de notre étude à la connaissance

- L'étiologie du genu valgum n'a pas d'influence significative sur le résultat final de l'OFV;
- La lame plaque Strélizia 95° constitue un matériel de choix pour la fixation des OFV et permet d'optimiser les résultats fonctionnels; l'OFV est une procédure difficile à régler avec précision;
- La correction finale de la déformation ne devait pas dépasser 3° degrés de genu varum.

## Conflits d'intérêts

Les auteurs ne déclarent aucun conflit d'intérêts.

## References

[1] Ankri J (2004). Problèmes économiques et sociaux posés par les affections de l'appareil locomoteur du sujet âgé. Rev Rhum..

[2] Antonescu DN (2000). L'Ostéotomie du Genou Est-elle Encore Indiquée dans la Gonarthrose. Acta Orthop Belg..

[3] Puddu G, Franco V, Cipolla M, Cerullo G, Gianni E (2006). Ostéotomie fémorale d'ouverture dans le genu valgum. La gonarthrose.

[4] Marin Morales LA, Gomez Navalon LA, Zorrilla Ribot P, Salido Valle JA (2000). Treatement of osteoarthritis of the knee with valgus deformity by means of varus osteotomy. Acta Orthop Belg..

[5] Aglietti P, Menchetti PP (2000). Distal femoral varus osteotomy in the valgus osteoarthritic knee. Am J Knee Surg..

[6] Mc Dermott AG, Finklestein JA, Farine I, Boynton EL, MacIntosh DL, Gross A (1988). Distal femoral varus osteotomy for valgus deformity of the knee. J Bone Joint Surg Am..

[7] Backstein D, Morag G, Hanna S, Safir O, Gross A (2007). Long-term follow-up of distal femoral varus osteotomy of the knee. J Arthroplasty..

[8] Langlais F, Lambotte J (1999). Ostéotomie du fémur distal. Encycl Med Chir Tech Chir Orthop Traumatol..

[9] Insall JN, Dorr LD, Scott RD, Scott WN (1989). Rationale of the Knee Society clinical rating system. Clin Orthop..

[10] Puddu G, Cipolla M, Cerullo G, Franco V, Giannì E (2010). Which osteotomy for a valgus knee?. Int Orthop..

[11] Zarrouk A, Bouzidi R, Karray B, Kammoun S, Mourali S, Kooli M (2010). Distal femoral varus osteotomy outcome: Is associated femoropatellar osteoarthritis consequential?. Orthop Traumatol Surg Res..

[12] Debette C, Parratte S, Maucort-Boulch D, Blanc G, Pauly V, Lustig S (2014). Adaptation française du nouveau score de la Knee Society dans l'arthroplastie de genou. Rev Chir Orthopédique Traumatol..

[13] Wang J-W, Hsu C-C (2006). Distal femoral varus osteotomy for osteoarthritis of the knee - Surgical technique. J Bone Joint Surg Am..

[14] Zilber S, Larrouy M, Sedel L, Nizard R (2004). Ostéotomie fémorale distale de varisation pour genuvalgum invalidant: Résultats à long terme et revue de la littérature. Rev Chir Orthopédique Réparatrice Appar Mot..

[15] Stähelin T, Hardegger F, Ward JC (2000). Supracondylar osteotomy of the femur with use of compression - Osteosynthesis with a malleable implant. J Bone Joint Surg Am..

[16] Puddu G, Vittorio E, Cipolla M, Cerullo G, Gianni E (2008). Opening wedge osteotomy of the distal femur in the valgus knee. Osteoarthritis of the knee..

[17] Insall JN, Ranawat CS, Aglietti P, Shine J (1976). A comparison of four models of total knee-replacement prostheses. J Bone Joint Surg Am..

[18] Franco V, Cerullo G, Cipolla M, Gianni E, Puddu G (2005). Osteotomy for osteoarthritis of the knee. Curr Orthop..

[19] Ouadih R, Berrada M, Elmrini A, Kharmaz M, Lahlou A, Wahbi S (2005). Genu Valgum (A propos de 16 cas). Rev Maroc Chir Orthop Traumato..

[20] Luna-Pizarro D, Moreno-Delgado F, De la Fuente-Zuno JC, Meraz-Lares G (2012). Distal femoral dome varus osteotomy: surgical technique with minimal dissection and external fixation. The Knee..

